# Correlation between estimated glucose disposal rate and in-stent restenosis following percutaneous coronary intervention in individuals with non-ST-segment elevation acute coronary syndrome

**DOI:** 10.3389/fendo.2022.1033354

**Published:** 2022-11-14

**Authors:** Chi Liu, Qi Zhao, Ziwei Zhao, Xiaoteng Ma, Yihua Xia, Yan Sun, Dai Zhang, Xiaoli Liu, Yujie Zhou

**Affiliations:** ^1^Department of Cardiology, Beijing Anzhen Hospital, Capital Medical University, Beijing, China; ^2^Beijing Institute of Heart Lung and Blood Vessel Disease, Beijing Key Laboratory of Precision Medicine of Coronary Atherosclerotic Disease, Clinical Center for Coronary Heart Disease, Capital Medical University, Beijing, China

**Keywords:** estimated glucose disposal rate, non-ST-segment elevation acute coronary syndrome, percutaneous coronary intervention, insulin resistance, in-stent restenosis

## Abstract

**Background:**

Insulin resistance (IR) is closely associated with in-stent restenosis (ISR) following percutaneous coronary intervention (PCI). Nevertheless, the predictive power of the newly developed simple assessment method for IR, estimated glucose disposal rate (eGDR), for ISR after PCI in individuals with non-ST-segment elevation acute coronary syndrome (NSTE-ACS) remains unclear.

**Methods:**

NSTE-ACS cases administered PCI in Beijing Anzhen Hospital between January and December 2015 were enrolled. The included individuals were submitted to at least one coronary angiography within 48 months after discharge. Patients were assigned to 2 groups according to ISR occurrence or absence. eGDR was derived as 21.16 - (0.09 * waist circumference [cm]) - (3.41 * hypertension) - (0.55 * glycated hemoglobin [%]). Multivariate logistic regression analysis and receiver operating characteristic (ROC) curve analysis were performed for evaluating eGDR’s association with ISR.

**Results:**

Based on eligibility criteria, 1218 patients were included. In multivariate logistic analysis, the odds ratios (ORs) of eGDR as a nominal variate and a continuous variate were 3.393 (confidence interval [CI] 2.099 - 5.488, P < 0.001) and 1.210 (CI 1.063 - 1.378, P = 0.004), respectively. The incremental effect of eGDR on ISR prediction based on traditional cardiovascular risk factors was reflected by ROC curve analysis (AUC: baseline model + eGDR 0.644 vs. baseline model 0.609, P for comparison=0.013), continuous net reclassification improvement (continuous-NRI) of -0.264 (p < 0.001) and integrated discrimination improvement (IDI) of 0.071 (p = 0.065).

**Conclusion:**

In NSTE-ACS cases administered PCI, eGDR levels show an independent negative association with increased ISR risk.

## Introduction

Although the popularization of second-generation drug-eluting stents (DESs) has largely decreased in-stent hyperproliferation, the incidence of in-stent restenosis (ISR) remains high, between 3% and 20%, which confirms coronary anatomical characteristics, patient indexes and surgical factors are highly correlated (1–3). The mechanism of ISR development is complex: besides vascular factors such as endothelial dysfunction, smooth muscle hyperplasia and inflammation (4–6), age, gender, hypertension, hyperlipidemia, diabetes and smoking are also considered risk factors for ISR (4, 7–10). Because of such complexity, accurate prediction and prevention of ISR has important clinical significance in improving prognosis in atherosclerotic cardiovascular disease (ASCVD) treated with stents.

Type 2 diabetes mellitus (T2DM) represents a major risk factor for ASCVD, which includes coronary heart disease, cerebrovascular disease and peripheral arterial disease (PAD), and also plays a key role in ISR (11). As an important pathogenetic mechanism of T2DM, insulin resistance (IR) has been shown to be correlated with the occurrence of ISR (12–14). IR measurement and assessment have attracted extensive attention recently. Hyperinsulinemic-euglycemic clamp is presently considered the gold standard for IR evaluation, but its wide clinical application is hampered by its high cost, time-consuming, complex and invasive characteristics. Using hyperinsulinemic-euglycemic clamp as a validation criterion, investigators established an estimated glucose disposal rate (eGDR) to enable the evaluation of insulin sensitivity in type 1 diabetes mellitus (T1DM) (15, 16). In the original study, waist-to-hip ratio (WHR), hypertension, and glycated hemoglobin (HbA1c) were included in the formula of eGDR. However, further studies have shown utilizing waist circumference (WC) in lieu of WHR for eGDR determination yields comparable results (15, 17). Patients with high eGDR have higher insulin sensitivity; conversely, low eGDR is associated with enhanced IR (18).

It was demonstrated that low eGDR independently predicts all-cause mortality in T2DM cases administered coronary artery bypass grafting (CABG) (19). Nevertheless, no studies have explored the relationship between eGDR and ISR. Therefore, we conducted the current work to investigate eGDR’s predictive value in ISR for individuals with non-ST-elevation acute coronary syndrome (NSTE-ACS) administered percutaneous coronary intervention (PCI).

## Materials and methods

### Study population

**Figure 1:**
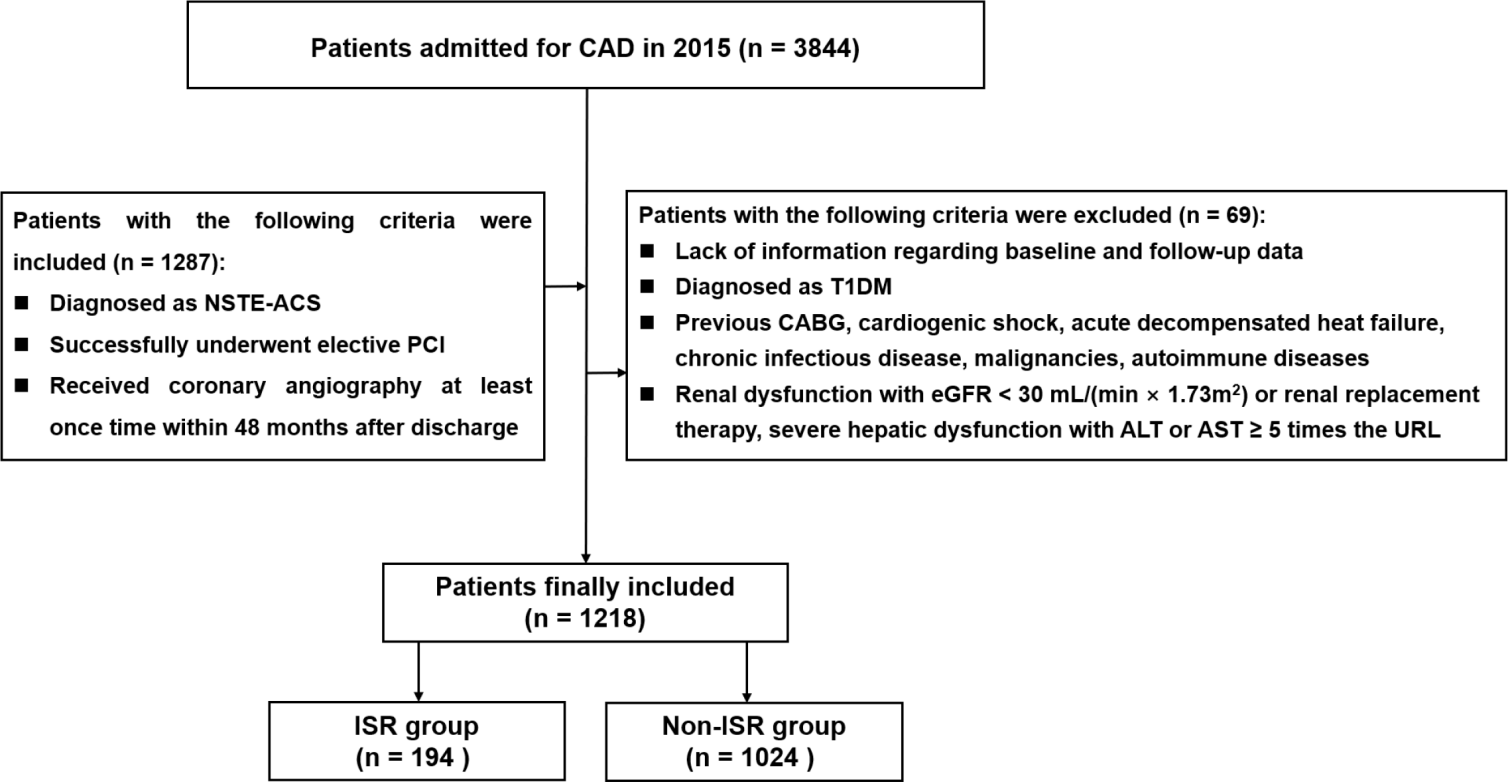
Flow diagram for the enrollment of study population. *CAD* coronary artery disease, *NSTE-ACS* non-ST-segment elevation acute coronary syndrome, *PCI* percutaneous coronary intervention, *T1DM* Type 1 Diabetes mellitus, *CABG* coronary artery bypass grafting, *eGFR* estimated glomerular filtration rate, *ALT* alanine transaminase, *AST* aspartate transaminase, *URL* upper reference limit, *ISR* in-stent restenosis.

This single-center observational trial enrolled individuals diagnosed with coronary artery disease (CAD) in Beijing Anzhen Hospital between January and December 2015. Inclusion criteria were: (1) diagnosis of NSTE-ACS (including on-ST-segment elevation myocardial infarction [NSTEMI] and unstable angina [UA]); (2) successful elective PCI; (3) coronary angiography performed at least once within 48 months after discharge. Relevant diagnostic criteria were based on the latest guidelines (20, 21). Exclusion criteria were: (1) missing baseline and/or follow-up data; (2) T1DM diagnosis; (3) history of CABG, cardiogenic shock, acute decompensated heat failure, chronic infectious disease, or cancer; (4) impaired kidney function, with estimated glomerular filtration rate (eGFR) below 30 mL/(min × 1.73 m^2^) or kidney replacement treatment; (5) serious liver dysfunction, with alanine transaminase and/or aspartate transaminase ≥ 5 times the respective upper reference limits. A total of 1218 individuals were finally included (**Figure 1**). The study was approved by the Hospital Clinical Research Ethics Committee and was conducted in accordance with the Helsinki Declaration.

### Coronary intervention and stenting

Coronary angiography, coronary stent implantation, and perioperative management were all performed by two experienced interventional cardiologists, with the implementation path and management process based on current guidelines (21). Cases underwent antiplatelet treatment, with loading doses of 300, 300 and 180 mg for aspirin, clopidogrel and ticagrelor, respectively, prior to interventional therapy. During the procedure, 100 IU/kg unfractionated heparin was also administered for anticoagulation to maintain an activated clotting time >300 seconds. Successful stent placement was considered with residual stenosis <20% in the target lesion, as assessed by visual inspection or quantitative coronary angiography, and grade-III anterior thrombolysis in myocardial infarction (TIMI) flow.

### Data collection and definitions

Demographic and clinical characteristics were recorded by hospital information center professionals. NSTE-ACS includes non-ST segment elevation myocardial infarction (NSTEMI) and unstable angina pectoris (UA), whose definitions refer to recognized guidelines (22). Diagnostic criteria for related diseases (T2DM, hypertension, dyslipidemia, stroke, and PAD) followed current guidelines (23–27). WC was measured by taking the distance of the midpoint line between the rib’s lowest point and the iliac crest’s upper border. Echocardiography-based diagnostic reports were evaluated and reviewed by two sonographers. Blood samples were collected after fasting for 8-12 hours and transported to the hospital’s testing center for testing of hematological and biochemical parameters. A variety of biochemical and hematological indicators were collected. The standard enzymatic method was used to determine triglyceride (TG), total cholesterol (TC) and high-density lipoprotein cholesterol (HDL-C). The homogeneous direct method was performed to determine low-density lipoprotein cholesterol (LDL-C). The enzymatic hexokinase technique was performed to detect fasting blood glucose (FBG). Other parameters and indicators were determined by the standard laboratory method in the central laboratory of the hospital. The synergy between PCI and taxus and cardiac surgery (SYNTAX) score was determined using a standard formula (http://www.syntaxscore.com).

The formula for calculating eGDR was as follows (15, 17, 28): eGDR = 21.16 - (0.09 * WC [cm]) - (3.41 * Hypertension [yes or no]) - (0.55 * HbA1c [%]).

### Definition and judgment of ISR

All the 1218 patients included in this study completed a 48-month follow-up period and underwent at least one coronary angiography in our hospital within 48 months of discharge. ISR was considered with a stenosis ≥ 50% in diameter within the stent or involving 5 mm proximal and distal to the stent (29). Quantitative coronary angiography was used to assess coronary stenosis. Similarly, angiographic findings and the presence of ISR were examined by two independent experienced cardiologists. Participants were assigned to the ISR and non-ISR groups, based on ISR status at 48 months.

### Statistical analysis

Participants’ baseline data were described by the following methods. Continuous data with normal and skewed distributions were described as mean ± standard deviation (SD) and median with 25^th^ and 75^th^ percentiles, respectively, and compared by the two-sample t-test and the Mann-Whitney U test, respectively. Nominal variables were described as number and percentage, and comparison used the chi-square, continuity-adjusted chi-square, or Fisher’s exact test.

Univariate logistic regression analysis was used to identify parameters associated with ISR. Baseline variables with significant associations in univariate analysis and those clinically significant for ISR development were further assessed by multivariable logistic regression analysis, excluding variates that may have collinearity. eGDR was evaluated as both nominal and continuous. Nominal variables were analyzed for the low and high eGDR groups, categorized based on median eGDR (lower eGDR [eGDR ≤ 6.92]; higher eGDR [eGDR > 6.92]). Odds ratio (OR) and 95% confidence interval (CI) were determined for each association. Four multivariable logistic regression models were built for assessing eGDR’s association with ISR. In Model 1, adjustment was made for age, sex and body mass index (BMI). Model 2 was adjusted for Model 1’s variables besides a history of smoking, previously diagnosed myocardial infarction (MI), a history of PCI and previously detected stroke. In Model 3, adjustment was made for Model 2’s variables in addition to TG, LDL-C, high-sensitivity C-reactive protein (hs-CRP), eGFR and left ventricular ejection fraction (LVEF). Model 4 was adjusted for Model 3’s variables as well as angiotensin-converting enzyme inhibitor/angiotensin receptor blocker (ACEI/ARB) at discharge, left main artery (LM) lesion, bifurcation, multi-vessel lesion, chronic total occlusion (CTO) lesion, SYNTAX score, complete revascularization and DES amount.

Subgroup analysis was performed after stratification by T2DM, adjusted for model 4 variates. The area under the receiver operating characteristic (ROC) curve (AUC) was obtained to assess eGDR’s predictive value in ISR. Net reclassification improvement (NRI) and integrated discrimination improvement (IDI) illustrated the incremental impact of introducing eGDR on the predictive ability of currently accepted risk models. The baseline model used for comparison included the following cardiovascular risk factors: age, sex, BMI, smoking history, family history of CAD, previously diagnosed MI, previously diagnosed PCI, previously detected stroke, hyperlipidemia, LVEF and SYNTAX score.

SPSS 26.0 and R 3.6.3 were utilized for data analysis, with two-sided P < 0.05 indicating statistical significance.

## Results

### Baseline patient features

Totally 1218 participants averaging 59.93 ± 8.90 years old were included, with a male proportion of 70.4% (n=858). Details of demographics, past medical history, laboratory tests, drug status and interventions for the non-ISR and ISR groups are presented in **Table 1**. In comparison with non-ISR cases, the ISR group showed elevated WC and higher rates of smoking history, drinking history, diabetes, hypertension, previous MI, and previous PCI. Regarding laboratory tests, ISR cases showed elevated FBG and HbA1c amounts, but reduced TC and LDL-C levels. For admission medication, patients with ISR had higher rates of dual antiplatelet therapy (DAPT), aspirin, P2Y12 inhibitors, β-blockers, statins, oral hypoglycemic agents (OHA) and insulin. For discharge medication, the rates of ACEI/ARB, OHA and insulin used were elevated in ISR cases. Regarding coronary angiography and PCI, ISR cases displayed elevated rates of bifurcation and SYNTAX score, but lower rates of complete revascularization. The mean length of stent was higher in the ISR group, but there was no difference in minimal stent diameter between the two groups. Baseline data grouped by median eGDR are presented in **Supplementary Table S1**

**Table 1:** Baseline characteristics of the study population based on ISR.

	Total population(n = 1218)	Non-ISR(n = 1024)	ISR(n = 194)	*P* value
Age, years	59.93 ± 8.90	59.88 ± 9.01	60.22 ± 8.32	0.627
Sex, male, n (%)	858 (70.4)	712 (69.5)	146 (75.3)	0.109
BMI, kg/m^2^	26.17 ± 3.18	26.12 ± 3.19	26.45 ± 3.13	0.184
WC, cm	91.51 ± 12.33	91.21 ± 12.46	93.11 ± 11.49	0.038
Heart rate, bpm	70.14 ± 10.34	70.03 ± 10.21	70.73 ± 11.02	0.386
SBP, mmHg	130.61 ± 16.70	130.54 ± 16.44	130.97 ± 18.07	0.386
DBP, mmHg	77.02 ± 9.94	76.95 ± 9.91	77.39 ± 10.12	0.572
Smoking history, n (%)	686 (56.3)	561 (54.8)	125 (64.4)	0.013
Drinking history, n (%)	290 (23.8)	233 (22.8)	57 (29.4)	0.047
Family history of CAD, n (%)	126 (10.3)	104 (10.2)	22 (11.3)	0.620
Medical history, n (%)				
Diabetes	432 (35.5)	333 (32.5)	99 (51.0)	< 0.001
Hypertension	787 (64.6)	643 (62.8)	144 (74.2)	0.002
Hyperlipidemia	1051 (86.3)	883 (86.2)	168 (86.6)	0.891
Previous MI	235 (19.3)	185 (18.1)	50 (25.8)	0.013
Previous PCI	190 (15.6)	144 (14.1)	46 (23.7)	0.001
Previous stroke	138 (11.3)	109 (8.9)	29 (2.4)	0.083
Previous PAD	170 (14.0)	135 (13.2)	35 (18.0)	0.073
Clinical diagnosis, n (%)				0.401
UA	1017 (83.5)	859 (83.9)	158 (81.4)	
NSTEMI	201 (16.5)	165 (16.1)	36 (18.6)	
Laboratory examinations				
TG, mmol/L	1.47 (1.04, 2.06)	1.47 (1.04, 2.08)	1.43 (1.03, 1.94)	0.450
TC, mmol/L	4.14 ± 1.02	4.18 ± 1.02	3.95 ± 1.01	0.004
LDL-C, mmol/L	2.52 ± 0.86	2.55 ± 0.85	2.39 ± 0.86	0.017
HDL-C, mmol/L	0.98 ± 0.23	0.98 ± 0.24	0.96 ± 0.20	0.144
hs-CRP, mg/L	1.28 (0.57, 3.32)	1.28 (0.55, 3.20)	1.29 (0.64, 3.52)	0.092
Creatinine, μmol/L	76.13 ± 17.10	76.05 ± 17.09	76.53 ± 17.15	0.722
eGFR, mL/(min × 1.73m^2^)	93.06 ± 20.39	92.87 ± 20.15	94.05 ± 21.66	0.459
Uric acid, μmol/L	346.60 ± 81.31	346.45 ± 82.22	347.40 ± 76.56	0.881
FBG, mmol/L	6.09 ± 1.74	107.96 ± 30.16	118.11 ± 36.13	< 0.001
HbA1c, mmol/mol	44.97 ± 12.72	44.35 ± 12.34	48.26 ± 14.14	< 0.001
LVEF, %	64.08 ± 6.48	64.13 ± 6.38	63.83 ± 6.95	0.557
Medication at admission, n (%)				
ACEI/ARB	285 (23.4)	234 (22.9)	51 (26.3)	0.300
DAPT	359 (29.5)	273 (26.7)	86 (44.3)	< 0.001
Aspirin	639 (52.5)	512 (50.0)	127 (65.5)	< 0.001
P2Y12 inhibitors	385 (31.6)	297 (29.0)	88 (45.4)	< 0.001
β-Blocker	268 (22.0)	209 (20.4)	59 (30.4)	0.002
Statins	361 (29.6)	292 (28.5)	69 (35.6)	0.049
OHA	220 (18.1)	169 (16.5)	51 (26.3)	0.001
Insulin	115 (9.4)	83 (8.1)	32 (16.5)	< 0.001
Medication at discharge, n (%)				
ACEI/ARB	868 (71.3)	714 (69.7)	154 (79.4)	0.006
DAPT	1217 (99.9)	1023 (99.9)	194 (100.0)	0.663
Aspirin	1218 (100.0)	1024 (100.0)	194 (100.0)	
P2Y12 inhibitors	1218 (100.0)	1024 (100.0)	194 (100.0)	
β-Blocker	1113 (91.4)	929 (90.7)	184 (94.8)	0.061
Statins	1192 (97.9)	1004 (98.0)	188 (96.9)	0.314
Ezetimibe	128 (10.5)	104 (10.2)	24 (12.4)	0.356
OHA	217 (17.8)	167 (16.3)	50 (25.8)	0.002
Insulin	112 (9.2)	81 (7.9)	31 (16.0)	< 0.001
Angiographic data, n (%)				
LM lesion	50 (4.1)	43 (4.2)	7 (3.6)	0.704
Bifurcation	243 (20.0)	193 (18.8)	50 (25.8)	0.027
Multi-vessel lesion	808 (66.3)	672 (65.6)	136 (70.1)	0.226
In-stent restenosis	70 (5.7)	55 (5.4)	15 (7.7)	0.195
Chronic total occlusion lesion	153 (12.6)	128 (12.5)	25 (12.9)	0.882
SYNTAX score	10.52 ± 5.29	10.38 ± 5.28	11.21 ± 5.26	0.047
Procedural information				
Minimal stent diameter, mm	2.86 ± 0.37	2.87 ± 0.36	2.83 ± 0.27	0.226
Mean length of stent, mm	22.33 ± 4.15	22.16 ± 4.02	23.19 ± 4.70	0.005
Target vessel territory, n (%)				
LM	31 (2.5)	24 (2.3)	7 (3.6)	0.305
LAD	784 (64.4)	660 (64.5)	124 (63.9)	0.886
LCX	413 (33.9)	356 (34.8)	57 (29.4)	0.146
RCA	532 (43.7)	450 (43.9)	82 (42.3)	0.666
Complete revascularization, n (%)	712 (58.5)	613 (59.9)	99 (51.0)	0.022
Number of DES	2.00 (1.00, 3.00)	2.00 (1.00, 3.00)	2.00 (1.00, 3.00)	0.698

ISR in-stent restenosis, BMI body mass index, WC waist circumference, SBP systolic blood pressure, DBP diastolic blood pressure, CAD coronary artery disease, MI myocardial infarction, PCI percutaneous coronary intervention, PAD peripheral artery disease, UA unstable angina, NSTEMI non-ST-segment elevation myocardial infarction, TG triglyceride, TC total cholesterol, LDL-C low-density lipoprotein cholesterol, HDL-C high-density lipoprotein cholesterol, hs-CRP high-sensitivity C-reactive protein, eGFR estimated glomerular filtration rate, FBG fasting blood glucose, HbA1c glycosylated hemoglobin A1c, LVEF left ventricular ejection fraction, ACEI angiotensin-converting enzyme inhibitor, ARB angiotensin receptor blocker, DAPT dual antiplatelet therapy, OHA oral hypoglycemic agents, LM left main artery, SYNTAX synergy between PCI with taxus and cardiac surgery, LAD left anterior descending artery, LCX left circumflex artery, RCA right coronary artery, DES drug-eluting stent.

### Predictive value of eGDR for ISR

Univariate analysis was performed for initially identifying factors associated with ISR (**Supplementary Table S2**). Based on univariate logistic regression analysis and clinically relevant risk factors, we screened variates and built four multivariate logistic regression models to measure eGDR’s predictive value in ISR. Whether defined as a nominal variate (with higher median eGDR as reference) or a continuous variate (per 1-unit decrease), eGDR had an independent predictive value across all 4 models. After fully adjusting for potential confounders in Model 4, ORs for eGDR as a nominal variate and a continuous variate were 3.393 (2.099-5.488) and 1.210 (1.063 - 1.378), respectively (**Table 2**).

**Table 2:** Association of eGDR with ISR in multivariate logistic regression analysis.

	As nominal variate^a^		As continuous variate^b^	
	OR (95% CI)	*P* value	OR (95% CI)	*P* value
Unadjusted	2.591 (1.866-3.598)	< 0.001	1.169 (1.087-1.256)	< 0.001
Model 1	2.983 (2.048-4.345)	< 0.001	1.218 (1.111-1.335)	< 0.001
Model 2	2.960 (2.019-4.339)	< 0.001	1.200 (1.094-1.315)	< 0.001
Model 3	3.019 (2.048-4.450)	< 0.001	1.200 (1.094-1.317)	< 0.001
Model 4	3.393 (2.099-5.488)	< 0.001	1.210 (1.063-1.378)	0.004

Model 1: adjusted for age, sex, BMI.

Model 2: adjusted for variates in Model 1 and smoking history, previous MI, previous PCI, previous stroke.

Model 3: adjusted for variates in Model 2 and TG, LDL-C, hs-CRP, eGFR, LVEF.

Model 4: adjusted for variates in Model 3 and ACEI/ARB at discharge, LM lesion, bifurcation, multi-vessel lesion, chronic total occlusion lesion, SYNTAX score, complete revascularization, number of DES.

^a^The OR was evaluated regarding the higher median of eGDR as reference.

^b^The OR was evaluated by per 1-unit decrease of eGDR.

eGDR estimated glucose disposal rate calculated, ISR in-stent restenosis, OR odds ratio, CI confidence interval.

**Figure 2:**
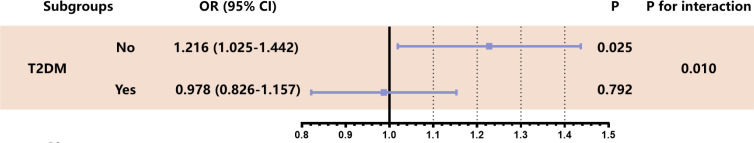
Stratified analysis of eGDR predicting ISR in T2DM subgroup. The analysis was adjusted for Model 4 except for variates applied for grouping. OR was evaluated by per 1-unit decrease of eGDR. *eGDR* estimated glucose disposal rate calculated, *ISR* in-stent restenosis, *OR* odds ratio, *T2DM* type 2 diabetes mellitus.

Subgroup analysis of the independent association between eGDR and ISR based on T2DM status was carried out (**Figure 2**). The results revealed eGDR’s predictive potential in ISR was higher in non-T2DM cases [OR (95%CI): T2DM no 1.216 (1.025-1.442) vs. T2DM yes 0.978 (0.826–1.157), P for interaction = 0.010].

### Incremental efficacy of eGDR for ISR prediction

**Figure 3:**
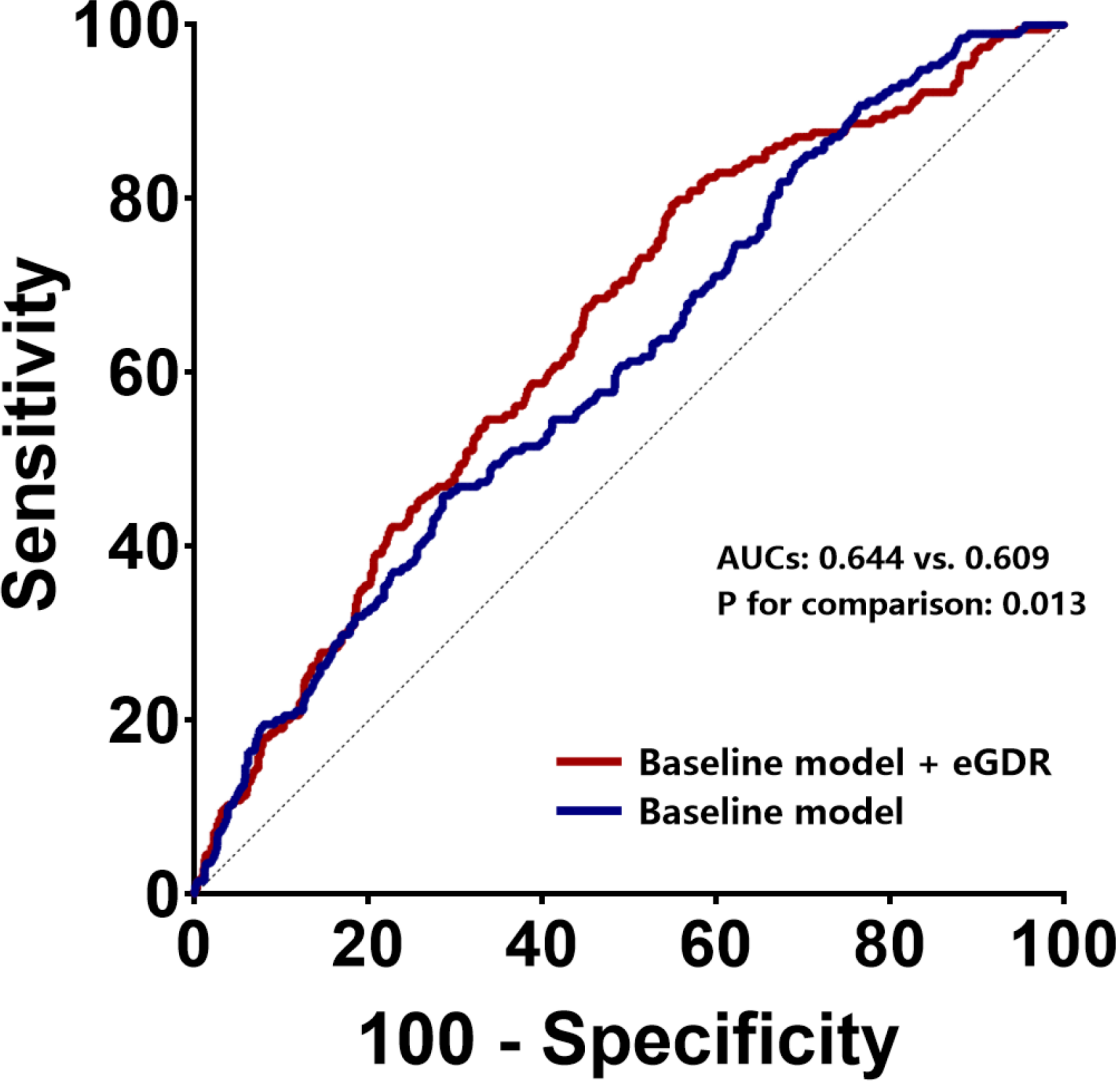
ROC curves to assess the predictive value of eGDR for ISR in general population. *ROC* receiver-operating characteristic, *eGFR* estimated glomerular filtration rate, *ISR* in-stent restenosis, *AUC* area under curve.

We established baseline models based on currently recognized cardiovascular risk factors as mentioned in Methods. Based on this model, addition of eGDR significantly enhanced its predictive power for ISR (AUCs of 0.644 and 0.609 for baseline model + eGDR and baseline model, respectively; P = 0.013) (**Table 3**, **Figure 3**). Estimation of continuous-NRI (-0.264, p < 0.001) also showed similar results, although IDI values (0.071, p = 0.065) were not significantly different (**Table 3**).

**Table 3:** Incremental effect of eGDR on ISR prediction by existing risk model in general population.

	ROC curve analysis				Continuous-NRI			IDI		
	AUC	95% CI	P value	P for comparison	Estimation	95% CI	P value	Estimation	95% CI	P value
Baseline model^a^	0.609	0.567-0.652	< 0.001	–	–	–	–	–	–	–
+ eGDR	0.644	0.603-0.685	< 0.001	0.013	-0.264	-0.294–0.234	< 0.001	0.071	-0.004-0.147	0.065

eGDR estimated glucose disposal rate, ISR in-stent restenosis, ROC receiver-operating characteristic, NRI net reclassification improvement, IDI integrated discrimination improvement, AUC area under curve, CI confidence interval.

a Baseline model includes age, sex, BMI, smoking history, family history of CAD, previous MI, previous PCI, previous stroke, hyperlipidemia, LVEF, SYNTAX score.

### Prediction of ISR by eGDR based on T2DM status

**Figure 4:**
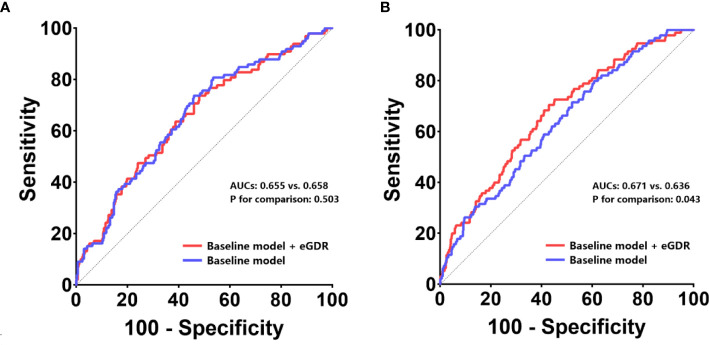
ROC curves to assess the predictive value of eGDR for ISR in populations with and without T2DM. The predictive values of the eGDR and baseline models were assessed in populations with T2DM **(A)** and without T2DM **(B)**. *ROC* receiver-operating characteristic, *eGFR* estimated glomerular filtration rate, *ISR* in-stent restenosis, *AUC* area under curve, *T2DM* type 2 diabetes mellitus.

In non-diabetic cases, eGDR showed an incremental effect similar to that of the general population, with AUCs of 0.671 and 0.636 for baseline model + eGDR and baseline model, respectively (P = 0.043); continuous-NRI was 0.091 (P < 0.001) and IDI was 0.081 (P = 0.103) (**Table 4**, **Figure 4B**). In contrast, in the diabetic population, addition of eGDR did not increase the predictive potential of the baseline model in ROC curve analysis (AUCs of 0.655 and 0.658 for baseline model + eGDR and baseline model, respectively; P = 0.503), and continuous-NRI (-0.021, P = 0.107) and IDI (-0.021, P = 0.394) differences were not statistically significant (**Table 4**, **Figure 4A**).

**Table 4:** Incremental effect of eGDR on ISR prediction by existing risk model in populations with and without T2DM.

	ROC curve analysis				Continuous-NRI			IDI		
	AUC	95% CI	P value	P for comparison	Estimation	95% CI	P value	Estimation	95% CI	P value
With T2DM										
Baseline model^a^	0.658	0.597-0.718	< 0.001	–	–	–	–	–	–	–
+ eGDR	0.655	0.593-0.716	< 0.001	0.503	-0.021	-0.047-0.005	0.107	-0.021	-0.068-0.026	0.394
Without T2DM										
Baseline model^a^	0.636	0.578-0.693	< 0.001	–	–	–	–	–	–	–
+ eGDR	0.671	0.615-0.728	< 0.001	0.043	0.091	0.056-0.126	<0.001	0.081	-0.016-0.178	0.103

eGDR estimated glucose disposal rate, ISR in-stent restenosis, ROC receiver-operating characteristic, NRI net reclassification improvement, IDI integrated discrimination improvement, AUC area under curve, CI confidence interval, T2DM type 2 diabetes mellitus.

a Baseline model includes age, sex, BMI, smoking history, family history of CAD, previous MI, previous PCI, previous stroke, hyperlipidemia, LVEF, SYNTAX score.

## Discussion

The present work firstly assessed eGDR’s association with ISR following PCI in CAD. The results revealed eGDR was independently and negatively associated with increased risk of ISR following PCI in NSTE-ACS; furthermore, eGDR improved the predictive ability of routine cardiovascular risk factors for ISR; moreover, the predictive value of eGDR for ISR was mainly reflected in patients without T2DM.

IR is the most important pathogenetic mechanism of diabetes and metabolic syndrome, with the main features including the following two aspects: decreased ability of insulin to induce glucose uptake and use; body compensation by enhanced insulin secretion for inducing hyperinsulinemia to stabilize blood sugar. Insulin resistance causes endothelial dysfunction, oxidative stress, and the activation of inflammatory responses, ultimately leading to the formation of atherosclerotic plaques (30). Currently, assessment techniques for insulin resistance mostly encompass two categories: direct assessment methods and simple surrogate assessment indicators. The hyperinsulinemic-euglycemic clamp and the insulin suppression test are both direct assessment methods for insulin resistance. By applying the hyperinsulinemic-euglycemic clamp, researchers confirmed that IR is tightly associated with coronary atherosclerotic heart disease, with a predictive role independent of other risk factors (31–33). For simple surrogate assessment indicators of IR, many clinical studies have used homoeostasis model assessment of insulin resistance (HOMA-IR) as an assessment method to explore the relationship between IR and cardiovascular disease (CVD), with consistent results. Indeed, IR is highly associated with atherosclerosis (34) and predicts CVD onset and poor prognosis in non-diabetic individuals (35–37). However, in clinical practice, fasting insulin levels are not routinely measured even in diabetics, let alone in individuals without diabetes. In addition, insulin measurement methods do not yield consistent data across laboratories, especially in case of low insulin levels. Therefore, researchers have proposed a variety of simpler alternative assessment indicators of insulin resistance, including triglyceride-glucose (TyG) index, triglyceride/high-density lipoprotein cholesterol (TG/HDL-C), visceral adiposity index (VAI) and lipid accumulation product (LAP), which are highly correlated with the incidence and prognosis of ASCVD (38–41). eGDR is also a simple surrogate measure of this type of IR.

It has long been admitted that diabetes could predict the occurrence of ISR (42, 43), and a study suggested that diabetes is the most effective predictor of ISR (44). In addition, a meta-analysis showed ISR incidence is markedly elevated in diabetic patients in comparison with non-diabetics (45). Therefore, diabetes can almost be considered the clearest risk factor for ISR. Previously, it was shown that IR is a common feature of CVD patients undergoing stent surgery, and an important marker of restenosis after PCI, with a deterioration process related to endothelial dysfunction, nitric oxide production disorders and activity defects (13). In recent years, studies applying HOMA-IR have confirmed that insulin resistance is highly correlated with ISR occurrence after PCI, representing an independent predictor of ISR (12, 14). In addition, a study using TyG as an evaluation index of IR found that TyG is independently and positively correlated with ISR risk following DES implantation in ACS patients (46).

As for eGDR, its associations with stroke incidence and mortality in T2DM patients have been demonstrated (47). In addition, eGDR was also shown to be closely related to elevated risk of all-cause mortality after CABG in T2DM patients, independent of other cardiovascular and metabolic risk factors (19). The above findings suggest that eGDR has great potential in predicting ASCVD prognosis and ISR events after PCI. This study clarified the predictive potential of eGDR for ISR occurrence post-PCI in NSTE-ACS cases, which is consistent with previous findings. The present work not only confirmed IR could predict ISR occurrence upon PCI in NSTE-ACS cases, but also revealed a new and effective indicator applicable for ISR prediction. The population of this study was mainly UA patients. On the one hand, we excluded patients who underwent primary PCI due to severe and complex disease as well as confounding factors that were difficult to adjust for. On the other hand, the patient data available to our research team came from general wards with relatively few NSTEMI patients. In data analysis, we attempted to include diabetes and related variates in the multivariate analysis, but after final adjustment, eGDR lost statistical significance in ISR prediction. Therefore, a subgroup analysis was carried out based on the diabetes status. The results revealed eGDR only had a predictive value in ISR for the non-diabetic subgroup. Furthermore, incremental effect analysis in the diabetes and non-diabetes groups was also consistent with the above subgroup analysis. This could explain the lack of significance for eGDR in models incorporating diabetes and associated variates. As mentioned above, IR assessed by various methods has important predictive value for CVD development in patients without diabetes. Although such finding is novel, we believe that eGDR can predict the adverse prognosis of CVD in non-diabetic patients. However, the results of the subgroup analysis in this study need further research to verify. It is certain that eGDR has the potential as a routine evaluation index of CVD cases, which requires further investigation in large prospective trials. In the era of widespread PCI treatment, there is a lack of simple and effective evaluation methods for long-term prognosis of patients. eGDR is expected to become an effective index to evaluate the ISR risk of patients after PCI and guide follow-up treatment. Finally, whether eGDR can really be used clinically as a powerful predictor of ISR after PCI needs to be assessed *via* comparison with other IR evaluation indicators.

There were some limitations in the present study that need to be further confirmed by more rationally designed studies. First, this was a single-center observational study of Chinese individuals, with unavoidable selection bias. Therefore, multi-center trials or even randomized controlled studies with larger samples and greater racial diversity are warranted to further clarify the current results. Additionally, because of the exclusion of patients undergoing emergency PCI, UA cases in this study cohort constituted the greatest part of all cases, and the current findings might not reflect the prognostic value of eGDR for ISR in NSTEMI. Furthermore, regarding repeat coronary angiography after discharge, ISR detection was not based on intracoronary imaging, and its accuracy was insufficient. Moreover, this work did not clarify the specific time when ISR occurred within 48 months after discharge and lacked short-term and long-term ISR analyses. In addition, this study did not compare the predictive value of eGDR and other IR evaluation methods on ISR.

## Conclusions

eGDR independently predicts ISR after PCI in NSTE-ACS cases and improves the predictive power of routine cardiovascular risk factors in ISR. Finally, eGDR’s predictive potential in ISR was mainly demonstrated in non-T2DM patients.

## Data availability statement

The raw data supporting the conclusions of this article will be made available by the authors, without undue reservation.

## Ethics statement

Written informed consent was obtained from the individual(s) for the publication of any potentially identifiable images or data included in this article.

## Author contributions

CL made substantial contributions to data collection, data analysis and manuscript writing. YZ and XL made substantial contributions to study design and intellectual direction. QZ, ZZ, XM, YX, YS, and DZ made contributions to data collection and analysis. All authors read and approved the final manuscript.

## Funding

The study was funded by Beijing Municipal Administration of Hospitals “Mission plan” (SML20180601) and the National Key Research and Development Program of China (2017YFC0908800).

## Conflict of interest

The authors declare that the research was conducted in the absence of any commercial or financial relationships that could be construed as a potential conflict of interest.

## Publisher’s note

All claims expressed in this article are solely those of the authors and do not necessarily represent those of their affiliated organizations, or those of the publisher, the editors and the reviewers. Any product that may be evaluated in this article, or claim that may be made by its manufacturer, is not guaranteed or endorsed by the publisher.

## Abbreviations

DES, drug-eluting stent; ISR, in-stent restenosis; ASCVD, atherosclerotic cardiovascular disease; T2DM, type 2 diabetes mellitus; PAD, peripheral arterial disease; IR, insulin resistance; eGDR, estimated glucose disposal rate; T1DM, type 1 diabetes mellitus; WHR, waist-to-hip ratio; HbA1c, glycosylated hemoglobin; WC, waist circumference; CABG, coronary artery bypass grafting; NSTE-ACS, non-ST-segment elevation acute coronary syndrome; PCI, percutaneous coronary intervention; CAD, coronary artery disease; NSTEMI, non-ST-segment elevation myocardial infarction; UA, unstable angina; eGFR, estimated glomerular filtration rate; TIMI, thrombolysis in myocardial infarction; TG, triglyceride; TC, total cholesterol; HDL-C, high-density lipoprotein cholesterol; LDL-C, low-density lipoprotein cholesterol; FBG, fasting blood glucose; SYNTAX, the synergy between PCI with taxus and cardiac surgery; SD, standard deviation; OR, odds ratio; CI, confidence interval; BMI, body mass index; MI, myocardial infarction; hs-CRP, high-sensitivity C-reactive protein; LVEF, left ventricular ejection fraction; ACEI, angiotensin-converting enzyme inhibitor; ARB, angiotensin receptor blocker; LM, left main artery; CTO, chronic total occlusion; ROC, receiver operating characteristic; AUC, area under curve; NRI, net reclassification improvement; IDI, integrated discrimination improvement; OHA, oral hypoglycemic agents; HOMA-IR, homoeostasis model assessment of insulin resistance; CVD, cardiovascular disease; TyG, triglyceride-glucose; HDL-C, high-density lipoprotein cholesterol; VAI, visceral adiposity index; LAP, lipid accumulation product

## Glossary

**Table:**

DES	drug-eluting stent
ISR	in-stent restenosis
ASCVD	atherosclerotic cardiovascular disease
T2DM	type 2 diabetes mellitus
PAD	peripheral arterial disease
IR	insulin resistance
eGDR	estimated glucose disposal rate
T1DM	type 1 diabetes mellitus
WHR	waist-to-hip ratio
HbA1c	glycosylated hemoglobin
WC	waist circumference
CABG	coronary artery bypass grafting
NSTE-ACS	non-ST-segment elevation acute coronary syndrome
PCI	percutaneous coronary intervention
CAD	coronary artery disease
NSTEMI	non-ST-segment elevation myocardial infarction
UA	unstable angina
eGFR	estimated glomerular filtration rate
TIMI	thrombolysis in myocardial infarction
TG	triglyceride
TC	total cholesterol
HDL-C	high-density lipoprotein cholesterol
LDL-C	low-density lipoprotein cholesterol
FBG	fasting blood glucose
SYNTAX	the synergy between PCI with taxus and cardiac surgery
SD	standard deviation
OR	odds ratio
CI	confidence interval
BMI	body mass index
MI	myocardial infarction
hs-CRP	high-sensitivity C-reactive protein
LVEF	left ventricular ejection fraction
ACEI	angiotensin-converting enzyme inhibitor
ARB	angiotensin receptor blocker
LM	left main artery
CTO	chronic total occlusion
ROC	receiver operating characteristic
AUC	area under curve
NRI	net reclassification improvement
IDI	integrated discrimination improvement
OHA	oral hypoglycemic agents
HOMA-IR	homoeostasis model assessment of insulin resistance
CVD	cardiovascular disease
TyG	triglyceride-glucose
HDL-C	high-density lipoprotein cholesterol
VAI	visceral adiposity index
LAP	lipid accumulation product
