# Supplementary material for: Correlation between estimated glucose disposal rate and in-stent restenosis following percutaneous coronary intervention in individuals with non-ST-segment elevation acute coronary syndrome
Source: Front Endocrinol (Lausanne). 2022 Nov 14;13:1033354. doi: 10.3389/fendo.2022.1033354 (PMC9702552; doi:10.3389/fendo.2022.1033354)
Supplement: Supplementary file 1 [file DataSheet_1.docx]

## Table S1. Baseline characteristics of the study population in two group based on median eGDR

|  | Lower eGDR  (≤ 6.92; n = 608) | Higher eGDR  (> 6.92; n = 610) | *P* value |
| --- | --- | --- | --- |
| Age, years | 59.84±8.77 | 60.02±9.03 | 0.721 |
| Sex, male, n (%) | 444 (73.0) | 414 (67.9) | 0.049 |
| BMI, kg/m^2^ | 27.73±2.81 | 24.63±2.86 | < 0.001 |
| WC, cm | 97.88±10.18 | 85.15±10.92 | < 0.001 |
| Heart rate, bpm | 71.28±10.60 | 69.00±9.96 | < 0.001 |
| SBP, mmHg | 132.50±16.78 | 128.73±16.41 | < 0.001 |
| DBP, mmHg | 77.97±10.35 | 76.08±9.43 | 0.001 |
| Smoking history, n (%) | 359 (59.0) | 327 (53.6) | 0.056 |
| Drinking history, n (%) | 152 (25) | 138 (22.6) | 0.330 |
| Family history of CAD, n (%) | 67 (11.0) | 59 (9.7) | 0.440 |
| Medical history, n (%) |  |  |  |
| Diabetes | 302 (49.7) | 130 (21.3) | < 0.001 |
| Hypertension | 591 (97.2) | 196 (32.1) | < 0.001 |
| Hyperlipidemia | 541 (89.0) | 510 (83.6) | 0.006 |
| Previous MI | 119 (19.6) | 116 (19.0) | 0.806 |
| Previous PCI | 100 (16.4) | 90 (14.8) | 0.415 |
| Previous stroke | 87 (14.3) | 51 (8.4) | 0.001 |
| Previous PAD | 23 (3.8) | 23 (3.8) | 0.991 |
| Clinical diagnosis, n (%) |  |  | 0.303 |
| UA | 501 (82.4) | 516 (84.6) |  |
| NSTEMI | 107 (17.6) | 94 (15.4) |  |
| Laboratory examinations |  |  |  |
| TG, mmol/L | 1.52 (1.09, 2.16) | 1.41 (0.97, 2.00) | 0.002 |
| TC, mmol/L | 4.09±1.03 | 4.19±1.01 | 0.079 |
| LDL-C, mmol/L | 2.50±0.86 | 2.54±0.85 | 0.323 |
| HDL-C, mmol/L | 0.94±0.21 | 1.01±0.25 | < 0.001 |
| hs-CRP, mg/L | 1.53 (0.70, 3.60) | 1.09 (0.46, 2.91) | < 0.001 |
| Creatinine, μmol/L | 77.42±17.41 | 74.84±16.69 | 0.008 |
| eGFR, mL/(min × 1.73m^2^) | 92.15±20.55 | 93.96±20.21 | 0.121 |
| Uric acid, μmol/L | 354.55±81.23 | 338.67±80.69 | 0.001 |
| FBG, mmol/L | 6.46±1.95 | 5.71±1.41 | < 0.001 |
| HbA1c, % | 6.59±1.28 | 5.94±0.93 | < 0.001 |
| LVEF, % | 63.99±6.54 | 64.17±6.41 | 0.633 |
| Medication at admission, n (%) |  |  |  |
| ACEI/ARB | 217 (35.7) | 68 (11.1) | < 0.001 |
| DAPT | 188 (30.9) | 171 (28.0) | 0.269 |
| Aspirin | 334 (54.9) | 305 (50.0) | 0.085 |
| P2Y12 inhibitors | 196 (32.2) | 189 (31.0) | 0.638 |
| β-Blocker | 158 (26.0) | 110 (18.0) | 0.001 |
| Statins | 178 (29.3) | 183 (30.0) | 0.782 |
| OHA | 164 (27.0) | 56 (9.2) | < 0.001 |
| Insulin | 83 (13.7) | 32 (5.2) | < 0.001 |
| Medication at discharge, n (%) |  |  |  |
| ACEI/ARB | 587 (96.5) | 281 (46.1) | < 0.001 |
| DAPT | 608 (100.0) | 609 (99.8) | 0.318 |
| Aspirin | 608 (100.0) | 610 (100.0) |  |
| P2Y12 inhibitors | 608 (100.0) | 610 (100.0) |  |
| β-Blocker | 575 (94.6) | 538 (88.2) | < 0.001 |
| Statins | 591 (97.2) | 601 (98.5) | 0.111 |
| OHA | 161 (26.5) | 56 (9.2) | < 0.001 |
| Insulin | 80 (13.2) | 32 (5.2) | < 0.001 |
| Angiographic data, n (%) |  |  |  |
| LM lesion | 25 (4.1) | 25 (4.1) | 0.991 |
| Bifurcation | 137 (22.5) | 106 (17.4) | 0.024 |
| Multi-vessel lesion | 441 (72.5) | 367 (60.2) | < 0.001 |
| In-stent restenosis | 35 (5.8) | 35 (5.7) | 0.989 |
| Chronic total occlusion lesion | 80 (13.2) | 73 (12.0) | 0.531 |
| SYNTAX score | 11.10±5.43 | 9.94±5.08 | < 0.001 |
| Procedural information |  |  |  |
| Minimal stent diameter, mm | 2.83±0.29 | 2.89±0.42 | 0.008 |
| Mean length of stent, mm | 22.19±4.19 | 22.46±4.11 | 0.252 |
| Target vessel territory, n (%) |  |  |  |
| LM | 14 (2.3) | 17 (2.8) | 0.592 |
| LAD | 382 (62.8) | 402 (65.9) | 0.263 |
| LCX | 221 (36.3) | 192 (31.5) | 0.072 |
| RCA | 288 (23.6) | 244 (40.0) | 0.010 |
| Complete revascularization, n (%) | 334 (54.9) | 378 (62.0) | 0.013 |
| Number of DES | 2.00 (1.00, 3.00) | 2.00 (1.00, 3.00) | 0.054 |

*eGDR* estimated glucose disposal rate, *BMI* body mass index, *WC* waist circumference, *SBP* systolic blood pressure, *DBP* diastolic blood pressure, *CAD* coronary artery disease, *MI* myocardial infarction, *PCI* percutaneous coronary intervention, *PAD* peripheral artery disease, *UA* unstable angina, *NSTEMI* non-ST-segment elevation myocardial infarction, *TG* triglyceride, *TC* total cholesterol, *LDL-C* low-density lipoprotein cholesterol, *HDL-C* high-density lipoprotein cholesterol, *hs-CRP* high-sensitivity C-reactive protein, *eGFR* estimated glomerular filtration rate, *FBG* fasting blood glucose, *HbA1c* glycosylated hemoglobin A1c, *LVEF* left ventricular ejection fraction, *ACEI* angiotensin-converting enzyme inhibitor, *ARB* angiotensin receptor blocker, *DAPT* dual antiplatelet therapy, *OHA* oral hypoglycemic agents, *LM* left main artery, *SYNTAX* synergy between PCI with taxus and cardiac surgery, *LAD* left anterior descending artery, *LCX* left circumflex artery, *RCA* right coronary artery, *DES* drug-eluting stent

## Table S2. Univariate logistic regression analysis investigating predictors of ISR

| Variables | OR | 95% CI | *P* value |
| --- | --- | --- | --- |
| Age, per 1 years | 1.004 | 0.987-1.022 | 0.627 |
| Gender, male as reference | 0.750 | 0.528-1.067 | 0.110 |
| BMI, per 1 kg/m^2^ | 1.033 | 0.985-1.084 | 0.184 |
| WC, per 1 cm | 1.013 | 1.000-1.025 | 0.049 |
| Heart rate, per 1 bpm | 1.006 | 0.992-1.021 | 0.385 |
| SBP, per 1 mmHg | 1.002 | 0.992-1.011 | 0.746 |
| DBP, per 1 mmHg | 1.004 | 0.989-1.020 | 0.571 |
| Smoking history | 1.495 | 1.087-2.056 | 0.013 |
| Drinking history | 1.412 | 1.004-1.988 | 0.048 |
| Family history of CAD | 1.131 | 0.695-1.843 | 0.620 |
| Diabetes | 2.162 | 1.585-2.949 | < 0.001 |
| Hypertension | 1.707 | 1.208-2.411 | 0.002 |
| Hyperlipidemia | 1.032 | 0.658-1.618 | 0.891 |
| Previous MI | 1.575 | 1.100-2.255 | 0.013 |
| Previous PCI | 1.899 | 1.306-2.763 | 0.001 |
| Previous stroke | 1.475 | 0.949-2.295 | 0.084 |
| Previous PAD | 1.450 | 0.964-2.180 | 0.075 |
| TG, per 1 mmol/L | 0.935 | 0.784-1.113 | 0.448 |
| TC, per 1 mmol/L | 0.790 | 0.672-0.929 | 0.004 |
| LDL-C, per 1 mmol/L | 0.792 | 0.635-0.960 | 0.018 |
| HDL-C, per 1 mmol/L | 0.630 | 0.316-1.256 | 0.189 |
| hs-CRP, per 1 mg/L | 1.018 | 0.995-1.042 | 0.117 |
| Creatinine, per 1 μmol/L | 1.002 | 0.993-1.011 | 0.721 |
| eGFR, per 1 mL/(min × 1.73m^2^) | 1.003 | 0.995-1.010 | 0.458 |
| Uric acid, μmol/L | 1.000 | 0.998-1.002 | 0.881 |
| FBG, per 1 mmol/L | 1.164 | 1.079-1.255 | < 0.001 |
| HbA1c, per 1% | 1.261 | 1.121-1.418 | < 0.001 |
| LVEF, per 1% | 0.993 | 0.970-1.017 | 0.557 |
| ACEI/ARB at admission | 1.204 | 0.847-1.711 | 0.300 |
| DAPT at admission | 2.191 | 1.598-3.003 | < 0.001 |
| Aspirin at admission | 1.896 | 1.376-2.611 | < 0.001 |
| P2Y12 inhibitors at admission | 2.032 | 1.486-2.780 | < 0.001 |
| Statins at admission | 1.384 | 1.001-1.913 | 0.049 |
| OHA at admission | 1.804 | 1.259-2.586 | 0.001 |
| Insulin at admission | 2.239 | 1.441-3.479 | < 0.001 |
| ACEI/ARB at discharge | 1.672 | 1.152-2.426 | 0.007 |
| Statins at discharge | 0.624 | 0.247-1.575 | 0.318 |
| OHA at discharge | 1.782 | 1.241-2.559 | 0.002 |
| Insulin at discharge | 2.214 | 1.417-3.459 | < 0.001 |
| LM lesion | 0.854 | 0.378-1.927 | 0.704 |
| Bifurcation | 1.495 | 1.045-2.138 | 0.028 |
| Multi-vessel lesion | 1.228 | 0.880-1.714 | 0.227 |
| In-stent restenosis | 1.476 | 0.816-2.671 | 0.198 |
| Chronic total occlusion lesion | 1.036 | 0.654-1.639 | 0.882 |
| SYNTAX score, per 1-point | 1.029 | 1.000-1.059 | 0.047 |
| Minimal stent diameter, mm | 0.766 | 0.497-1.180 | 0.226 |
| Mean length of stent, mm | 1.061 | 1.023-1.101 | 0.002 |
| LM treatment | 1.560 | 0.662-3.672 | 0.309 |
| LAD treatment | 0.977 | 0.710-1.345 | 0.886 |
| LCX treatment | 0.781 | 0.559-1.091 | 0.147 |
| RCA treatment | 0.934 | 0.685-1.274 | 0.666 |
| Complete revascularization | 0.699 | 0.513-0.951 | 0.023 |
| Number of DES, per 1 DES | 0.992 | 0.877-1.123 | 0.901 |

*BMI* body mass index, *WC* waist circumference, *SBP* systolic blood pressure, *DBP* diastolic blood pressure, *CAD* coronary artery disease, *MI* myocardial infarction, *PCI* percutaneous coronary intervention, *PAD* peripheral artery disease, *TG* triglyceride, *TC* total cholesterol, *LDL-C* low-density lipoprotein cholesterol, *HDL-C* high-density lipoprotein cholesterol, *hs-CRP* high-sensitivity C-reactive protein, *eGFR* estimated glomerular filtration rate, *FBG* fasting blood glucose, *HbA1c* glycosylated hemoglobin A1c, *LVEF* left ventricular ejection fraction, *ACEI* angiotensin-converting enzyme inhibitor, *ARB* angiotensin receptor blocker, *DAPT* dual antiplatelet therapy, *OHA* oral hypoglycemic agents, *LM* left main artery, *SYNTAX* synergy between PCI with taxus and cardiac surgery, *LAD* left anterior descending artery, *LCX* left circumflex artery, *RCA* right coronary artery, *DES* drug-eluting stent
